# Bone loss in hepatitis B virus-infected patients can be associated with greater osteoclastic activity independently of the retroviral use

**DOI:** 10.1038/s41598-021-89486-9

**Published:** 2021-05-13

**Authors:** Renata Dessordi, Ligia Moriguchi Watanabe, Mariana Palma Guimarães, Elen Almeida Romão, Ana de Lourdes Candolo Martinelli, Rodrigo de Carvalho Santana, Anderson Marliere Navarro

**Affiliations:** 1 Department of Food and Nutrition, Faculty of Pharmaceutical Sciences, State University of São Paulo – UNESP, Endereço: Avenida Bandeirantes, 3900. Monte Alegre, Ribeirão Preto, São Paulo 14049-900 Brazil; 2 Department of Clinical Medicine, Ribeirão Preto Medical School, University of São Paulo - FMRP/USP, São Paulo, Brazil

**Keywords:** Gastroenterology, Hepatology, Liver, Liver diseases

## Abstract

Nucleoside/nucleotide analogs such as tenofovir, have been used as long-term therapy for the treatment of hepatitis B and side effects such as the reduction in bone mineral density have been associated with their use. To determine the relationships between bone, hormonal, biochemical, and mineral parameters in patients with hepatitis B treated with nucleoside/nucleotide antiviral. A cross-sectional study was conducted with 81 adult patients with chronic hepatitis B infection. Dual-energy X-ray absorptiometry (DXA) was performed to assess bone mineral density. Biochemical analyses were performed for osteocalcin, deoxypyridinoline, parathyroid hormone, vitamin D, IGF-1, TSH, testosterone, estradiol, FSH, transaminases, urea, creatinine, calcium, serum and urinary phosphorus, magnesium, and FGF-23, body composition was performed by DXA. Participants, both gender, were divided according to the use of antiretrovirals: Group1: 27 inactive virus carriers without medication; Group2: 27 patients using tenofovir; and Group3: 27 patients using lamivudine or entecavir. DXA readings diagnosed osteopenia in the lumbar spine for 7.4% of individuals in Group1, 15% in Group2, and 3.7% in Group3. For all groups, we observed normal values in bone formation markers, osteocalcin levels as well as parathyroid hormone, insulin growth factor 1, and FGF-23. In all groups, we found increased levels of urinary deoxypyridinoline, a bone resorption marker. Increased levels in the bone resorption markers indicated a high resorptive activity of bone tissue. These data suggested high resorption activity of bone tissue in hepatitis B virus-infected patients independent of the use of antiretrovirals.

## Introduction

Hepatitis B is one of the most common chronic infections and constitutes an important global public health problem^1^. It is estimated that 2 billion people have already been infected and, about 350 to 400 million people have chronic hepatitis B virus infection (VHB)^2^. In Brazil, 718,837 cases of hepatitis B were reported, among acute and chronic infections, in the period from 1999 to 2017. Between 2000 and 2011, 9659 deaths related to hepatitis B were reported in the country^3^.

Currently, the treatment of hepatitis B virus infection is performed with medications belonging to two main groups: immunomodulatory agents (interferons) and nucleoside/nucleotide analogs (ANN). In the latter group, the most frequently used medications are included, with emphasis on entecavir and tenofovir^2,4^.

Tenofovir is a highly effective adenosine monophosphate ocyclic nucleotide analog in the treatment of hepatitis B^5,6^. However, the use of this medicine may interfere with bone mineral density7. The bone toxicity mechanism is unclear, suggesting three potential mechanisms that can lead to bone changes: preferential absorption by osteoclasts (altering gene expression and resulting in increased bone resorption), uptake by osteoblasts (altering gene expression and decreased bone formation), and uptake by osteoclasts and osteoblasts (alteration of gene expression of both cell types and finally, the balance between resorption and bone formation, resulting in a bone loss). Tenofovir is a phosphonate, that has preferential uptake by the bone cells, thus increasing the probability of cellular stress. Also, renal tubule dysfunction may occur resulting in hypophosphatemia and abnormalities in vitamin D metabolism^7–9^.

Data on the effects of tenofovir in patients with hepatitis B who are not infected with HIV are scarce and there are still many gaps in knowledge about changes in bone metabolism resulting from chronic HBV infection and by the use of antiretroviral drugs^10,11^. In this context, the objective of this study was to determine the relationships between bone, hormonal, biochemical, and mineral parameters in patients with hepatitis B treated with nucleoside/nucleotide antiviral.

## Casuistry and methods

### Ethical considerations

The research was approved by the Research Ethics Committee of the Clinical Hospital of Ribeirao Preto Medical School (HCFMRP-USP), opinion no. 1.497.995 and in the Brazilian Registry of Clinical Trials under the registry: RBR-6f4 × 7p. All patients signed the informed consent form before the beginning of the study. All methods were performed by the relevant guidelines and regulations established by the Research Ethics Committee of the Ribeirão Preto Medical School/FMRP-USP.

### Sample size

For the calculation of the sample size, the variable bone mineral density of the hip was considered, considered a marker used in studies that evaluate bone metabolism. Bone mineral density values were used in individuals with hepatitis B using tenofovir, and in patients not using this medication. The values found were 0.87 ± 0.14 and 0.86 ± 0.08 g/cm^2^, respectively^12^. For the variance, the largest variance found between the groups was taken (SPSS Statistics version 22.0).

Adopting a significance level of 0.05 and 0.80 of test power, the following equation was used to calculate the sample size^13^.

$$ n = \frac{{2\sigma^{2} (Z_{\alpha /2} + Z_{\beta } )^{2} }}{{(\mu_{1} - \mu_{2} )^{2} }}, $$

where *µ*_1_ and *µ*_21_ are the averages for each group. n = 81 participantes.

Results

- Test power = 80% and significance level 5%.

### Casuistry

The study was conducted with adult patients of both genders, with chronic hepatitis B virus infection in follow-up at the Hepatitis Outpatient Clinic of Ribeirao Preto Clinical Hospital. Overall 81 patients were included, and they were divided into three groups: 1—27 patients without medication, 2—27 patients using tenofovir, 3—27 patients using lamivudine and entecavir.

Inclusion criteria: confirmed diagnosis of chronic hepatitis B virus infection, age older than 30 years and less than 50 years, use of tenofovir for at least 12 months for Group 2, use of other ANNs (entecavir and lamivudine) for at least 12 months for Group 3, and no previous use of ANNs for Group 1.

Exclusion criteria: use of calcium and vitamin D supplements in the last year of inclusion in the study, cirrhosis CHILD B or C score, obesity or malnutrition, type 1 or 2 diabetes, use of medications to treat osteoporosis, participants with a history of thyroidectomy, renal failure, concomitant infection by HIV, concomitant infection by hepatitis C virus, use of corticosteroids in doses greater than or equal to 1 mg/kg body weight for more than 14 days before inclusion in the study, as well as during the course of the study, a rheumatologic disease in activity, women in menopause or with secondary amenorrhea, and women using hormone replacement therapy.

### Study design

A cross-sectional observational study was conducted that evaluated the prevalence of reduced bone mineral density in patients using tenofovir compared with individuals using lamivudine/entecavir and without antiretroviral drugs. After the selection, the participants participated in 2 meetings: The first one had the signature of the informed consent form; and the second one had the evaluation of bone mineral density through bone densitometry examination, collection of blood and urine samples to determine biochemical parameters and weight and height measurements.

### Evaluation of mineral bone density

Bone densitometry examination, DXA (Dual Energy X-ray Absorptiometry) was performed to evaluate the bone mineral density of the following bone sites: total hip, femoral neck, and lumbar spine using the apparatus Hologic QDR 4500A scanner; Hologic Inc., Waltham, MA, USA. Bone mineral density assessment was performed according to the classifications proposed by the World Health Organization (WHO)^14^. Thus, the criterion for classifying bone densitometry was defined after recruitment. Thus, as the volunteers were younger than 50 years of age, they were classified according to the Z-score.

### Markers involved in bone metabolism

For biochemical analysis, peripheral venous blood was collected after 12 h of fasting in the morning. For the collection of the urine sample on the second morning, the participant was given a sterile universal collector bottle, which was kept in an environment with low light. After collection, the blood samples were centrifuged.

Parathormonium (PTH), luteinizing hormone (LH), follicle-stimulating hormone (FSH), testosterone, estradiol, and insulin-like growth hormone 1 (IGF-1) were determined by using the IMMULITE 1000 immunochemical method (Siemens Healthineers, Erlangen, Germany). Albumin, creatinine, and urea were measured by the colorimetric method, using Labtest kits.

The determination of fibroblast growth factor 23 (FGF 23) and 25-hydroxyvitamin D was performed by the chemiluminescence immunoassay (CLIA) method, using the LIAISON test in the DIASORIN The 4000th Series analyzer. The determination of urinary deoxypyridinoline and osteocalcin was performed by the enzyme immunoassay method (ELISA), using the Quidel and Diasource Immuno Assays S.A. kits, respectively.

Total calcium, phosphorus, and magnesium were measured by the colorimetric method. Urinary phosphorus dosage was performed with a mass spectrometer (DRC-ICP-MS ELAN DRCII, Perkin Elmer, Sciex, Norwalk, CT, USA).

The estimate of the glomerular filtration rate (eTFG) was calculated by using the Chronic Kidney Disease Epidemiology Collaboration (CKD-EPI) equation.

eTGF = 141 × min (Scr/k, 1)a × max(Scr/k, 1) − 1.209 × 0.993age × 1.018 [for women] − 1.159 [for afro-descendants], where:

Scr is serum creatinine; k is 0.7 for female patients and 0.9 for male patients; a is equal to − 0.329 for female patients and − 0.411 for male patients, min indicates the minimum value of Scr/k or 1; and max indicates the maximum value of Scr/k^15–17^.

### Anthropometric evaluation

The weight, height, and body mass index (BMI) of the volunteers were measured. The body composition (lean and fat mass) was analyzed by the bone densitometry apparatus (DXA).

### Statistical analysis

Exploratory data analysis was performed for a global view of the characteristics of the variables obtained. The mean and standard deviation statistics were calculated. After the data distribution analysis, Shapiro–Wilk tests were performed to test the equality between the groups.

One-way ANOVA variance analysis test with Greenhouse–Geisser correction was applied and Tukey's post-test was used for multiple comparisons between the groups to verify significant differences (*p* ≤ 0.05). The student t-test was applied to compare two independent variables. Pearson's correlation test was performed by groups to quantify the association between two quantitative variables.

Multivariate linear regression analysis was performed by groups to evaluate the influence of the independent variable on the dependent variable, for those that presented significance in the correlation test. The software used for statistical analysis was SPSS Statistics version 22.0.

## Results

Table 1 presents the general characteristics of patients with chronic hepatitis B virus infection according to the study group. The mean age of the participants showed no difference (*p* = 0.13). The anthropometric and body composition parameters of weight, body mass index, fat mass, and muscle mass were the same for the three groups. There was no difference for the three groups regarding the duration of infection and time of exposure to medication and a higher viral load was observed for Group 1 compared with Groups 2 and 3 (*p* = 0.001). Bone densitometry showed no difference in the following parameters: lumbar spine, total hip, and femoral neck. Osteopenia was observed in 14.8% of Group 2 at the lumbar spine bone site.

**Table 1:** General characteristics of patients with chronic hepatitis B virus infection in use or not of antiretrovirals.

Variables	Group 1 Without antiretrovirals	Group 2 Tenofovir	Group 3 Lamivudine/Entecavir	*p* value
n	27	27	27	
Men (%)	40.7	50	48.1	–
Women (%)	59.2	50	51.8	–
Age (years)	39.9 ± 7.2	41.5 ± 8.4	44.1 ± 6.8	0.130
Weight (kg)	74.1 ± 15.4	72.9 ± 12.2	71.8 ± 15.8	0.842
BMI (kg/m^2^)	27.5 ± 5.1	26.5 ± 4.7	23.9 ± 4.8	0.782
eGFR, ml/min/1.73 m^2^	105 ± 9,6	102.5 ± 9.1	104.6 ± 7.2	0.701
**Body composition**				
Body fat (%)	35.1 ± 11.7	34.8 ± 12.8	34.1 ± 8.7	0.940
Lean Mass (%)	66.6 ± 11.1	66.5 ± 12.1	68 ± 9.4	0.847
**Chronic hepatitis B virus infection**				
Duration of infection in years	8.7 ± 3.6	11 ± 4.6	8.9 ± 3.8	0.07
Medication exposure time in years	–	6.7 ± 3.3	6.1 ± 13.6	0.06
Viral load (log)	2.8 ± 1.1^a^	1.25 ± 1.7^b^	1.54 ± 1.9^b^	0.001
Viral load undetectable (%)	7.4%	50%	40.9%	–
**BMD (g/cm**^**2**^**)**				
Lumbar spine Z score	1.221 ± 0.19	1.145 ± 0.14	1.251 ± 0.16	0.068
Osteopenia [n (%)]	2 (7.4)	4 (14.8)	2 (7.4)	–
Osteoporosis [n(%)]	–	–	1 (3.7)	–
Total hip Z score	1.062 ± 0.14	1.005 ± 0.12	1.069 ± 0.14	0.210
Osteopenia [n (%)]	–	1 (3.7)	2 (7.4)	–
Osteoporosis [n(%)]	–	–	–	–
Femoral neck Z score	1.039 ± 0.13	0.961 ± 0.22	1.030 ± 0.16	0.231
Osteopenia [n (%)]	–	2 (7.4)	1 (3.7)	–
Osteoporosis [n(%)]	–	–	1 (3.7)	–

ANOVA one way and student *t* test for independent samples (medication exposure time in years). Values are expressed as mean ± standard deviation. Different letters *p* ≤ 0.05. eGFR: estimation of glomerular filtration rate; BMD: bone mineral density.

Table 2 shows the results related to the serum and urinary biochemical parameters of all study groups. Analysis of the bone resorption marker, urinary deoxypyridinoline, revealed increased indexes for all groups (above 7.4 nmol/mmol). The values of osteocalcin and parathormone were within normal limits with higher levels for Group 2 (*p* = 0.01) when compared with Groups 1 and 3. The vitamin D dosages presented desirable values, being higher for Groups 2 and 3 compared with Group 1 (*p* = 0.01). The parameters FGF-23 and IGF-1 showed normal levels for all groups and no difference between them. Regarding the dosage of minerals in blood and urine, there was also no difference between the groups and their values were adequate.

**Table 2:** Serum and urinary biochemical parameters of patients with chronic hepatitis B virus infection in use or not of antiretrovirals.

Variables	Group 1 Without antiretrovirals	Group 2 Tenofovir	Group 3 Lamivudine/Entecavir	*p* value	Reference values
n	27	27	27		
DPD (nmol/mmol)	8.09 ± 2.98	9.23 ± 2.4	8.29 ± 2.5	0.295	2.3–7.4
Osteocalcin (ng/ml)	7.3 ± 3.95^a^	10.6 ± 5.67^b^	7.50 ± 3.85^ab^	0.025	< 2–21
PTH (pg/ml)	35.1 ± 16.0^ab^	43.2 ± 25.0^b^	28.0 ± 9.5^a^	0.011	10–65
IGF-1 (ng/ml)	191.5 ± 115.4	145.1 ± 69.2	137.1 ± 91.9	0.236	101–267
FGF-23 (pg/ml)	62.2 ± 24.7	54 ± 14.1	49.1 ± 19.2	0.168	23.2–95.4
25 OH vitamin D (ng/ml)	27.1 ± 7.1^a^	32.8 ± 9.2^b^	32.8 ± 7.9^b^	0.015	> 20
Total calcium (mg/dl)	9.23 ± 0.48	9.08 ± 0.42	9.17 ± 0.33	0.539	8.8–11
Phosphorus (mg/dl)	3.97 ± 0.64	3.66 ± 0.52	3.64 ± 0.61	0.086	3–7
Magnesium (mg/dl)	2.19 ± 0.18	2.21 ± 0.19	2.23 ± 0.18	0.686	1.9–2.5
Urinary phosphorus (mg/l)	538,6 ± 317,9	652,6 ± 349,2	487,9 ± 273,7	0.133	400–1300
GOT (U/l)	24.5 ± 10.3	24.0 ± 6.14	24.4 ± 9.2	0.982	5–40
GPT (U/l)	28.2 ± 18.4	27.7 ± 16.0	30.1 ± 20.1	0.876	7–56
Creatinine (mg/dl)	0.87 ± 0.1	0.90 ± 0.2	0.96 ± 0.2	0.375	0.6–1.2
Urea (mg/dl)	27.8 ± 7.9	28.6 ± 6.9	30.5 ± 8.0	0.413	10–40
Albumin (g/dl)	4.3 ± 0.5	4.3 ± 0.3	4.5 ± 0.3	0.099	3.5–4.7

ANOVA one way. Values are expressed as mean ± standard deviation. Different letters *p* ≤ 0.05. DPD, deoxypyridinoline; PTH, parathormone; IGF-1, insulin-like growth factor-1; FGF-23, fibroblast growth factor 23; GOT, glutamic-oxaloacetic transaminase; GPT, glutamic-pyruvic transaminase. FGF-23 n = 16–17 per group.

There was no difference in the concentrations of the hormones testosterone, LH, FSH, and estradiol and all participants presented values within the reference parameters. The glomerular filtration rate was within the normal range for all groups and the creatinine, urea, albumin, and liver transaminases tests were also adequate.

A correlation between IFG-1, urinary phosphorus, and magnesium was observed for Group 1. In Group 2 there was a correlation between osteocalcin and FGF-23 and for Group 3 between BMI, osteocalcin, and total calcium for the lumbar spine bone site (Table 3). Regarding the femoral neck parameter (Table 4), there was a correlation between BMI for Group 1 and for Group 3 between medication time, BMI, fat mass, and osteocalcin. The bone mineral density of the hip showed a correlation between BMI and osteocalcin for Group 1, and Group 3 between BMI and magnesium (Table 5).

**Table 3:** Correlation by groups between biochemical markers, body composition and viral parameters with bone mineral density of the lumbar spine in patients with chronic hepatitis B virus infection in use or not of antiretrovirals.

Variables	Lumbar spine					
	Group 1 Without antiretrovirals	*p* value	Group 2 Tenofovir	*p* value	Group 3 Lamivudine/Entecavir	*p* value
Age (years)	0.075	0.709	− 0.214	0.292	0.189	0.344
Viral load (log)	− 0.044	0.019	0.156	0.434	0.062	0.758
Medication time (years)	–	–	− 0.162	0.428	− 0.259	0.190
Vitamin D (ng/ml)	0.092	0.645	0.368	0.064	0.002	0.988
BMI (kg/m^2^)	0.071	0.723	0.247	0.222	0.369	0.05*
Body fat (kg)	0.016	0.933	0.123	0.547	0.173	0.387
Lean Mass (kg)	0.0004	0.998	0.109	0.594	0.051	0.798
IGF-1 (ng/ml)	0.373	0.05*	0.155	0.448	0.010	0.957
DPD (nmol/mmol)	− 0.194	0.363	− 0.131	0.540	0.011	0.955
Osteocalcin (ng/ml)	− 0.108	0.613	− 0.526	0.008*	− 0.535	0.007*
Total Calcium (mg/dl)	− 0.231	0.244	0.261	0.188	0.253	0.002*
Phosphorus (mg/dl)	− 0.119	0.552	− 0.097	0.630	0.156	0.437
Magnesium (mg/dl)	− 0.026	0.05*	0.304	0.122	0.315	0.108
Urinary phosphorus (mg/l)	0.135	0.05*	0.266	0.179	0.002	0.988
PTH (pg/ml)	0.170	0.395	0.075	0.708	− 0.030	0.879
FGF-23 (pg/ml)	0.225	0.401	− 0.680	0.002*	0.205	0.427

Parameters with statistical differences are given in bold.

Pearson correlation, significance level *p* ≤ 0.05 indicated by *. DPD, deoxypyridinoline; PTH, parathormone; IGF-1, insulin-like growth factor-1; FGF-23, fibroblast growth factor 23; BMI, body mass index. FGF-23 n = 16–17 per group.

**Table 4:** Correlation by groups between biochemical markers, body composition and viral parameters with bone mineral density of the femoral neck in patients with chronic hepatitis B virus infection in use or not of antiretrovirals.

Variables	Femoral neck					
	Group 1 Without antiretrovirals	*p* value	Group 1 Without antiretrovirals	*p* value	Group 1 Without antiretrovirals	*p* value
Age (years)	− 0.144	0.570	0.232	0.253	− 0.006	0.744
Viral load (log)	− 0.254	0.209	0.273	0.176	− 0.071	0.724
Medication time (years)	–	–	− 0.122	0.550	− 0.470	0.013*
Vitamin D (ng/ml)	0.156	0.435	0.128	0.524	− 0.042	0.831
BMI (kg/m^2^)	0.393	0.042*	0.092	0.645	0.604	0.0008*
Body fat (kg)	0.058	0.773	0.046	0.818	0.466	0.014*
Lean Mass (kg)	0.004	0.982	− 0.032	0.872	− 0.266	0.179
IGF-1 (ng/ml)	0.086	0.668	0.010	0.960	− 0.041	0.837
DPD (nmol/mmol)	− 0.205	0.335	− 0.138	0.518	− 0.066	0.757
Osteocalcin (ng/ml)	− 0.070	0.744	− 0.261	0.217	− 0.558	0.004*
Total Calcium (mg/dl)	− 0.113	0.581	− 0.211	0.300	0.215	0.279
Phosphorus (mg/dl)	− 0.064	0.752	− 0.031	0.877	0.197	0.323
Magnesium (mg/dl)	− 0.268	0.185	0.080	0.690	0.310	0.115
Urinary phosphorus (mg/l)	− 0.107	0.602	0.077	0.700	− 0.221	0.266
PTH (pg/ml)	− 0.082	0.689	− 0.006	0.972	− 0.288	0.145
FGF-23 (pg/ml)	0.074	0.783	− 0.304	0.234	0.162	0.532

Parameters with statistical differences are given in bold.

Pearson correlation, significance level *p* ≤ 0.05 indicated by *. DPD, deoxypyridinoline; PTH, parathormone; IGF-1, insulin-like growth factor-1; FGF-23, fibroblast growth factor 23; BMI, body mass index. FGF-23 n = 16–17 per group.

**Table 5:** Correlation by groups between biochemical markers, body composition and viral parameters with bone mineral density of the total hip in patients with chronic hepatitis B virus infection in use or not of antiretrovirals.

Variables	Total hip					
	Group 1 Without antiretrovirals	*p* value	Group 1 Without antiretrovirals	*p* value	Group 1 Without antiretrovirals	*p* value
Age (years)	0.131	0.513	− 0.135	0.509	− 0.004	0.980
Viral load (log)	− 0.262	0.186	0.336	0.092	0.126	0.529
Medication time (years)	–	–	0.066	0.746	− 0.325	0.097
Vitamin D (ng/ml)	0.127	0.525	0.172	0.399	0.092	0.647
BMI (kg/m^2^)	0.482	0.010*	0.133	0.516	0.572	0.001*
Body fat (kg)	0.057	0.774	0.053	0.796	0.343	0.079
Lean Mass (kg)	− 0.014	0.943	− 0.043	0.833	− 0.136	0.497
IGF-1 (ng/ml)	0.005	0.978	0.049	0.811	− 0.012	0.949
DPD (nmol/mmol)	− 0.159	0.457	0.042	0.845	− 0.164	0.442
Osteocalcin (ng/ml)	− 0.135	0.528	− 0.263	0.212	− 0.571	0.003*
Total Calcium (mg/dl)	0.061	0.759	− 0.096	0.638	0.295	0.134
Phosphorus (mg/dl)	− 0.063	0.752	0.206	0.311	0.153	0.444
Magnesium (mg/dl)	− 0.233	0.241	0.029	0.887	0.392	0.043*
Urinary phosphorus (mg/l)	− 0.030	0.880	0.146	0.475	− 0.108	0.590
PTH (pg/ml)	− 0.019	0.922	− 0.339	0.089	− 0.296	0.132
FGF-23 (pg/ml)	− 0.027	0.918	− 0.406	0.105	0.347	0.171

Parameters with statistical differences are given in bold.

Pearson correlation, significance level *p* ≤ 0.05 indicated by *. DPD, deoxypyridinoline; PTH, parathormone; IGF-1, insulin-like growth factor-1; FGF-23, fibroblast growth factor 23; BMI, body mass index. FGF-23 n = 16–17 per group.

For linear regression, the bone mineral density sites (lumbar spine, femoral neck, and total hip) and the BMI and viral load were used as independent variables. By analyzing the determination coefficients (R2): Table 6, it was possible to observe that the BMI showed a greater influence on the bone mineral density of the femoral neck and total hip for Groups 1 and 3 and the viral load variable for Group 1 and 3 was related to the bone mineral density of the lumbar spine.

**Table 6:** Multiple linear regression models by groups for the lumbar spine (model I), femoral neck (model II) and total hip (model III) of patients with chronic hepatitis B virus infection using or not antiretroviral drugs.

Model	Parameters	Estimate (beta)	SQ	*p* value	R^2^
I	**G1**				0.22
	BMI (kg/m^2^)	0.005	0.813	0.42	
	Viral load (log)	− 0.081	− 2.586	0.01	
	**G2**				0.12
	BMI (kg/m^2^)	0.009	1.549	0.13	
	Viral load (log)	0.22	1.340	0.19	
	**G3**				0.14
	BMI (kg/m^2^)	0.006	0.420	0.67	
	Viral load (log)	0.010	1.970	0.05	
II	**G1**				0.25
	BMI (kg/m^2^)	0.011	2.491	0.01	
	Viral load (log)	− 0.030	− 1.801	0.08	
	**G2**				0.04
	BMI (kg/m^2^)	0.005	0.557	0.58	
	Viral load (log)	0.02	1.010	0.32	
	**G3**				0.36
	BMI (kg/m^2^)	0.020	3.706	0.001	
	Viral load (log)	− 0.003	− 0.255	0.80	
III	**G1**				0.35
	BMI (kg/m^2^)	0.015	3.238	0.003	
	Viral load (log)	− 0.047	− 2.101	0.04	
	**G2**				0.04
	BMI (kg/m^2^)	0.005	0.557	0.58	
	Viral load (log)	0.027	1.015	0.32	
	**G3**				0.35
	BMI (kg/m^2^)	0.017	3.528	0.001	
	Viral load (log)	0.012	0.942	0.35	

Statistical difference: *p* ≤ 0.05. BMI, body mass index; G1, without antiretrovirals; G2, tenofovir group; G3, lamivudine/entecavir group.

## Discussion

This study investigated the possible changes in bone and mineral metabolism influenced by the use of antiretroviral drugs in patients with chronic hepatitis B virus infection, and it found changes in the marker of bone resorption for all groups, suggesting that viral infection can lead to bone changes regardless of the use of drugs.

The majority of the participants of this research presented age younger than 50 years and body weight within the normal range. Researchers demonstrate that advanced age, low or excess corporal weight are factors associated with the increased risk for the development of bone alterations, such as in osteoporosis^17,18^. A study conducted in HIV-positive patients that evaluated the association between BMI and body weight showed that overweight and underweight patients were more likely to develop changes in bone mineral density^19,20^.

In evaluating patients with chronic hepatitis B, the presence of infection was associated with an increased risk of bone alterations. Also, studies suggest that bone loss in chronic viral infections is the result of cumulative and time-dependent interactions between the patient's classic risk factors for the development of osteoporosis, such as viral load, associated inflammation, and use of antiretroviral drugs^7,21,22^. In this study, a low percentage of changes in bone mineral density was observed for the three groups. This fact may be associated with the non-inclusion of decompensated cirrhosis, adequate hormone levels, and normal glomerular filtration rate^17,23,24^. It is also important to mention that the findings of Gill et al^19^ were similar to this study, because they also found a low prevalence of osteopenia/osteoporosis in patients with chronic hepatitis B virus infection without severe liver complications, whether or not tenofovir is used.

Also, it is known that the viral infection itself and the use of antiretrovirals contributes to the development of systemic inflammation, which can result in early aging of tissues and metabolic bone changes^25,26^.

The evaluation of minerals (calcium, serum, and urinary phosphorus and, magnesium) showed that the participants of this study presented these parameters within the normal range. Correlation analysis revealed an association between magnesium, calcium, and urinary phosphorus levels in bone mineral density at the lumbar spine sites for Groups 1 and 3, and magnesium levels in hip bone mineral density for Group 3. Studies show that changes in phosphorus, calcium, and magnesium levels may affect bone formation and resorption, as well as hepatitis B virus infection itself, use of antiretroviral drugs, and consequently, systemic inflammation^27–29^.

Regarding the bone formation marker, osteocalcin, all groups presented adequate values, but urinary deoxypyridinoline levels (bone resorption marker) were increased, showing the existence of a marked bone remodeling activity. It is suggested that the increase in deoxypyridinoline in all groups may be associated with a viral infection, inflammatory status, and antiretroviral therapy as previously mentioned^27–29^.

Studies suggest that increased PTH value favors bone resorption, and reduced vitamin D impairs its role as an inhibitor in PTH production, decreasing calcium absorption in the intestinal tract, thus causing dysfunction in bone metabolism^30,31^. In this study, PTH and vitamin D concentrations were within normal ranges. In a study by Nguyen et al.^32^ in patients with chronic hepatitis B, it was found that the use of tenofovir or entecavir for a period of 12 months was not associated with the reduction in vitamin D levels. Similar evidence for this study found no alteration in the levels of this vitamin associated with the use of antiretrovirals.

Research shows that IGF-1 is related to the regulation of bone metabolism. This hormone is synthesized in the tissues and the skeleton is one of its largest reservoirs: therefore, the reduction in serum levels is associated with an increased risk of fractures^33^. In this study, IGF-1 levels were within the normal range, but it is important to mention that there was a between the levels of this hormone and BMD of the lumbar spine for Group 1. This fact can be explained by the higher viral load of this group, which did not use antiretroviral therapy; according to studies, viral infection may lead to increased risk of changes in biochemical parameters related to bone health^7,21,22^.

Regarding the performance of TDF in the kidney, the concentrations of serum and urinary phosphorus and the levels of the hormone FGF-23 were evaluated. There are studies in the literature that indicate that the use of TDF for approximately 7 years is safe and effective and that adverse renal effects occur in patients with a predisposition to renal diseases or comorbidities. However, other studies point out that prolonged use of TDF can lead to deregulation of phosphorus levels^13,34^. In this study, no changes were observed in the renal functions and concentrations of serum and urinary phosphorus in patients with a mean use of tenofovir for 6.7 ± 3.3 years. The hormone FGF-23 showed normal values for all groups and studies reveal that the elevation in this hormone is associated with increased excretion of phosphate by the kidneys and interference in the conversion of vitamin D to its active form (calcitriol)^35–37^. It is important to mention that the correlation analysis revealed that there is an association between levels of FGF-23 and bone mineral density of the lumbar spine for Group 2, confirming the data in the literature that suggests that higher levels of FGF-23 may lead to an increased risk in the development of bone alterations through the influence on phosphate and vitamin D metabolism^38^.

## Conclusion

The results regarding the biochemical parameters related to bone health suggest that the presence of chronic hepatitis B virus infection leads to an increased risk of changes in bone health regardless of the use of tenofovir, lamivudine, or entecavir. The increase in the bone resorption marker may suggest high resorption activity of bone tissue in patients with chronic hepatitis B virus infection regardless of the use of antiretroviral drugs. However further studies are needed to confirm these findings.

Studies about bone metabolism and how it can be affected by factors such as chronic infections and medications continue to evolve and pose a major challenge to better understand the physiology of bone loss. Therefore, further studies are needed to correlate changes in bone metabolism and fracture risk by evaluating a tenofovir use time of more than seven years to prevent or minimize changes in bone mineral density in patients with chronic hepatitis B virus infection.
